# Obtunding Dentin

**Published:** 1906-03-15

**Authors:** Ellison Hillyer


### OBTUNDING DENTIN.

BY ELLISON HILLYER, D.D.S.

The following is an extract from a report made to the New York State Dental Society, by Dr. Ellison Hillyer, of Brooklyn, He wrote several dentists and asked them the following questions?

(1) What method do you use to obtund sensitive dentine?

(2) Are you using any of the various new syringes intended for the above-stated purpose, and if so, with what results?

(3) How do you explain the phenomenon of thorough desentization of the dentin?

(4) In your opinion, does the cocain solution absolutely enter the pulp, and if so, do you anticipate any evil results therefrom?

(5) How does the action of cocain introduced in this manner compare with the action of cocain used by cataphoresis?

DR. C. N. JOHNSON, Chicago: I use desiccation, preceded by 95 percent carbolic acid in cases where the desiccation causes pain. I also use carbolic acid on a pellet of cotton, driving the agent into the dentin with a heated instrument applied to the cotton, and this followed by dryness. I use the Knowles obtunder with good effect in some cases, but most of all I use keenly sharp instruments, with the utmost delicacy of touch, and this latter, though very old and very hackneyed, is after all the best and most logical solution of the problem. If the profession generally would develop sufficient adroitness in the manipulation of instruments we would not hear so much about the terrors of sensitive dentine.

DR. R. OTTOLENGUI, New York: I am still fogy enough to like hot air and sharp instruments. Once in a while I get a little benefit with cocain pressure used exactly as it is for removing pulps. I am not using any of the various syringes. Such experiments as I made with one was not sufficiently satisfactory to make it of much value to me. I am quite confident that with the powerful syringes used, cocain solutions are forced into the pulp, and I certainly will expect in the near future to hear of trouble or in the distant future to find those pulps having pulp-nodules.

Cocain introduced with a syringe is badly injected into the pulp; by cataphoresis the cocain is separated electrically into its component ions.

DR. J. D. PATTERSON, Kansas City: I have two methods, first, the dehydration method, and second, cocain pressure method.

In dehydration, desensitization is accomplished by the use of hot air which removes the water from the protoplasm contained in the dentinal canaliculi, so that sensation is not conveyed to the pulp as when the tubules are filled with incompressible water (80 percent). In the cocain method for desensitization the cocain acts as an analgesic by virtue of its anesthetic effect, and by constriction of the vascular pulp tissue and protoplasmic contents of the dentinal tubules.

I believe the cocain does sometimes enter the pulp when used to obtund sensitive dentin, but often relief is obtained by the action of the cocain on the contents of the tubule alone. I prefer that the pulp shall not be penetrated by the cocain unless the pulp is to be obliterated. Whether the pulp is prejudiced if penetrated by cocain I do not know, but because I fear it may be I prefer dehydration methods, for experience has taught that little danger to the pulp comes by drying the contents of the tubules.

DR. WESTON A. PRICE, Cleveland: I use chiefly cataphoresis for extreme sensitiveness, local application of cocain in campho-phenique, etc., for slight sensitiveness, and, if pulp is to be removed for bridge abutment or otherwise, pressure anesthesia with cocain.

The high pressure syringe is generally effective, sometimes painful or slow. I am afraid to use it where I do not intend removing the pulp.

Cocain produces anesthesia of the dentin or pulp, or both, but not essentially the latter to desensitize the former.

The cocain solution may or may not enter the pulp according to the relation of time and propelling force to the resistance, and the unfavorable effect will depend upon the quantity and local conditions.

A larger quantity is introduced which is not so active per unit, nor so safe as when applied cataphorically. One-fourth of a milliampere of current will require about forty minutes to carry one-hundreth of a grain of cocain into a tooth. Under good conditions many times that amount in a second can be forced in with a pressure syringe, with danger of mechanically producing a lesion of the pulp tissue.

DR. JOHN S. MARSHALL, U. S. Army: The dental surgeons of the United States Army are not provided with cataphoric outfiits nor with the various syringes intended for the above purpose. We are therefore obliged to depend upon other and simpler means of mitigating the pain produced by excavating a sensitive carious cavity. I am in the habit of adopting one of the following methods:

First, a dry cavity—secured by the rubber dam and warm air—and a sharp, clean cutting bur run at high speed and manipulated with a *light hand*. Second, a moist cavity, kept constantly flooded with a stream of hot water—as hot as the patient can bear without pain—and the use of a sharp, clean cutting bur, etc., as above.

From my knowledge of and experience with cocain, I am of the opinion that when this drug is employed and forced into the tubuli, either by electric or mechanical pressure, thorough anesthesia of the dental pulp is produced. A partial or incomplete anesthesia may be produced in those dentinal fibers occupying the tubuli through which the cocain is traveling toward the pulp, but it is impossible to produce a complete anesthesia of the whole area of the dentin without first producing anesthesia of the dental pulp. There is no doubt in my mind that to produce complete anesthesia of the whole area of the dentin, the cocain must enter the pulp, but I can see no reason for "anticipating evil results therefrom" any more than that evil results should follow cocain anesthesia of the nasal mucous membrane or of the conjunctiva when employed for operations in these localities.

DR. HART J. GOSLEE, Chicago: For the purpose of obtunding sensitive dentine I use only dehydration with alcohol and hot air, and such anodynes as chloroform, menthol, creasote, oil of cloves or carbolic acid, used in conjunction with a sharp bur.

I am not now using any of the syringes designed for the purpose of forcing solutions of cocain into the tooth, for the reason that the amount of pressure cannot thus be easily controlled, and that any excess of pressure may produce trouble as a result of the deleterious influences which may be caused by forcing the ptomains contained in the decalcified dentin, or the cocain itself, into the apical area.

In my opinion the "phenomena" may be accounted for in the fact that "desensitization of the dentin" may only obtain as a direct result of more or less complete anesthetization of the pulp.

Cocain does enter the pulp, and does in a measure displace the normal fluids thereof. This may and often does produce evil results because of the fact that poisonous ptomains may be forced ahead of the cocain solution, or because of the further fact that cocain is itself accredited as being a protoplasmic poison, either of which may prove such a source of irritation as to result in subsequent trouble.

The action in so far as producing anesthesia is concerned is the same in cataphoresis as with the high pressure syringe, but in the "electric osmosis" of cataphoresis the electrolytic action of the current would break up or destroy the ptomains and thus lessen the possibilities of subsequent trouble therefrom.

DR. M. L. RHEIN, New York: I do not confine myself to any one means of obtunding sensitive dentin. I still use the cataphoric application of cocain, and together with that I have been using Myers' hypodermic syringe. The action of the latter appears to me to be identical with the effect produced by cataphoresis. I have yet to discover the first bad effect on the pulp from either of them.

In this connection, I might say that I do not use either of these agents in a number of so-called cases of sensitive dentin which can be readily controlled by the use of sharp burs and the inculcation of the principles of relaxation on the part of the patient.

DR. N. C. LEONARD, Nashville: The only method I have found satisfactory for obtunding sensitive dentin is complete desiccation and the use of sharp instruments. The sliding of a dull instrument on a sensitive surface causes much greater pain than the cutting of a sharp one.

Thorough desensitization of the dentin, in my opinion, can only be caused by anesthesia of the pulp. Therefore, I think the cocain solution would necessarily have to enter the pulp to produce this condition. I should anticipate frequent evil results from such treatment and for this reason would hesitate to adopt it except in cases where the pulp is to be removed.

DR. A. R. STARR, New York: I rely principally on methods of dehydration and cauterization to obtund sensitive dentin, and practice methods of accomplishing those results in conjunction with the use of sharp instruments. I do not expect much effect from local anesthetics unless the cavity is near the pulp.

In the methods I use (dehydration and cauterization), I believe that the abstraction of moisture interferes in some way with the transmission of sensation by the dentinal fibrils—how, I cannot explain, unless it be that those fibrils cannot perform the function of complete transmission of sensation in the absence of the normal amount of moisture. Cauterization, we may assume, destroys the superficial portion of the fibrils without destroying their integrity throughout. Just how far such cauterants as zinc chlorid, silver nitrate or carbolic acid affect the fibrils I do not know, but presume that being coagulants and not very penetrating they act only superficially.

DR. FRANK L. PLATT, San Francisco: I believe I have tried every method of obtunding sensitive dentin of which I have ever heard, both local and systemic, and have found all of them occasionally successful; even hot air works very nicely in some cases. I am now depending entirely on forcing a cocain solution into the dentin. I am using the Meyers dental obtunder, and in perhaps fifty cases in which I have used it I have yet to record a failure. I think it will always succeed if the pit drilled in the dentin is of the right size, is cut with a sharp, cleaning bur running in a true handpiece, if the syringe is in good order and the pressure kept up evenly for from one to three minutes by the watch (not by guess), the time depending on the density of the dentin and the degree of anesthesia required.

I think the dentin owes its sensitiveness to prolongations from the odontoblasts or from the nerve tissue of the pulp itself, extending into the dentinal tubuli, and this being the case anything which will obtund sensation anywhere will obtund sensitive dentin if properly applied, and all the dentin may be obtunded if the application is made to penetrate deeply enough.

The cocain solution must enter the pulp if the pressure is continued long enough, for I have removed pulps without pain in cases where the pit drilled into the dentin did not penetrate the pulp chamber, while in other cases I have found the dentin sensitive beyond the zone of anesthesia where the intention was simply to excavate a shallow cavity and the pressure had been continued but a short time.

My experience with cataphoresis was short and unsatisfactory, either from faulty apparatus or faulty manipulation, or both, though I bought two outfits and tried hard to use them. The only case in which I was entirely successful was one in which a patient complained of extreme sensitiveness in a tooth in which I found the canals (after half an hours' application of the cataphoric current), carefully filled with gutta-percha. So I do not feel competent to compare the two methods. I may say that so far I have noticed no systemic effect of the cocain in using the Myers obtunder.

DISCUSSION.

DR. F. E. HOWARD: I will say that I have used Dr. Myers' syringe and obtunding formula for about a year with great satisfaction. I do not believe the body of the pulp is ordinarily anesthetized, although it could be by long continued application. From all that I have learned I believe the anesthetic is brought in contact with the pulp, makes its impression on the periphery, and its influence is reflected back through other tubuli. Dr. Myers holds to this opinion, and he has done much experimenting. I have never had any deleterious effects follow its action on the pulp and do not anticipate such. I have never heard of a case that could be attributed directly to its action.

DR. H. P. GOULD: On making inquiries of a number of dentists as to the strength of solution used in the obtunding syringe, I am told by some that they use no known percentage, simply dissolving a few crystals in just enough water to carry the cocain. The solvent point of cocain is 0.48, which means that a certain given quantity of water will dissolve twice its weight of cocain. In other words that the saturated solution of cocain which may be used if made up in that way may be a 200 percentage solution.

A topical application of 10 or 20 percent cocain, applied by means of cotton, with the excessed squeezed out, will cause anesthesia of the mucous membrane lasting from thirty minutes to one hour and a half. Is it not most natural to suppose that a thorough anesthetization of the pulp with a solution of equal strength will cause an anesthesia of the pulp (which has no surrounding anastomotic nourishment), would last much longer? Considered from this standpoint what will be the result when a stronger solution is used?

To do permanent damage to a pulp under these conditions we will not need to force into it any ptomains from the decalcified dentin.

While cocain anesthesia lasts it means that the nutritive supply is cut off, and with the nutritive supply denied to any other part of the body for one hour and a half or longer, would you not naturally look for a speedy death of the tissue? I would say most emphatically, do not use cocain under pressure in a tooth, having no means of knowing the depth it will penetrate, unless you desire to extirpate the pulp.—*Cosmos.*

